# Are fetal growth impairment and preterm birth causally related to child attention problems and ADHD? Evidence from a comparison between high-income and middle-income cohorts

**DOI:** 10.1136/jech-2015-206222

**Published:** 2016-01-14

**Authors:** Elizabeth Murray, Rebecca Pearson, Michelle Fernandes, Iná S Santos, Fernando C Barros, Cesar G Victora, Alan Stein, Alicia Matijasevich

**Affiliations:** 1 Section of Child and Adolescent Psychiatry, Department of Psychiatry, Oxford University, Oxford, UK; 2 School of Social and Community Medicine, University of Bristol, Bristol, UK; 3 Nuffield Department of Obstetrics & Gynaecology, Oxford Maternal & Perinatal Health Institute, Green Templeton College, University of Oxford, Oxford, UK; 4 Postgraduate Program in Epidemiology, Federal University of Pelotas, Pelotas, Brazil; 5 Postgraduate Program in Health and Behavior, Catholic University of Pelotas, Pelotas, Brazil; 6 Department of Preventive Medicine, School of Medicine, University of São Paulo, São Paulo, Brazil

**Keywords:** Cohort studies, MATERNAL & CHILD CG, PERINATAL EPIDEMIOLOGY

## Abstract

**Background:**

Cross-cohort comparison is an established method for improving causal inference. This study compared 2 cohorts, 1 from a high-income country and another from a middle-income country, to (1) establish whether birth exposures may play a causal role in the development of childhood attention problems; and (2) identify whether confounding structures play a different role in parent-reported attention difficulties compared with attention deficit hyperactivity disorder (ADHD) diagnoses.

**Methods:**

Birth exposures included low birth weight (LBW), small-for-gestational age (SGA), small head circumference (HC) and preterm birth (PTB)). Outcomes of interest were attention difficulties (Strengths and Difficulties Questionnaire, SDQ) and ADHD (Development and Well-Being Assessment, DAWBA). Associations between exposures and outcomes were compared between 7-year-old children from the Avon Longitudinal Study of Parents and Children (ALSPAC) in the UK (N=6849) and the 2004 Pelotas cohort in Brazil (N=3509).

**Results:**

For attention difficulties (SDQ), the pattern of association with birth exposures was similar between cohorts: following adjustment, attention difficulties were associated with SGA (OR=1.59, 95% CI 1.20 to 2.19) and small HC (OR=1.64, 95% CI 1.11 to 2.41) in ALSPAC and SGA (OR=1.35, 95% CI 1.04 to 1.75) in Pelotas. For ADHD, however, the pattern of association following adjustment differed markedly between cohorts. In ALSPAC, ADHD was associated with LBW (OR=2.29, 95% CI 1.09 to 4.80) and PTB (OR=2.33, 95% CI 1.23 to 4.42). In the Pelotas cohort, however, ADHD was associated with SGA (OR=1.69, 95% CI 1.02 to 2.82).

**Conclusions:**

The findings suggest that fetal growth impairment may play a causal role in the development of attention difficulties in childhood, as similar associations were identified across both cohorts. Confounding structures, however, appear to play a greater role in determining whether a child meets the full diagnostic criteria for ADHD.

## Introduction

The associations between fetal growth impairment or preterm birth (PTB) and childhood attention problems have been reported in a number of studies.^1–3^ There is some evidence to suggest, however, that these associations may be an artefact of confounding by social and health factors.^4^ ^5^ Both fetal growth and PTB, as well as attentional problems, are related to socioeconomic factors and linked to family adversity and obstetric care.^6^ To date, there have been no comparisons of these associations between high-income and low-and-middle-income samples, where different confounding structures may be expected.^7^

The current study aimed to help determine whether the association may be causal, by comparing two different cohorts, one from the UK, a high-income country (the Avon Longitudinal Study of Parents and Children (ALSPAC)), and another from Brazil, a middle-income country (the 2004 Pelotas birth cohort study (Pelotas)). In addition to beginning 13 years apart, it is likely that different confounding structures exist within these populations.^7^ Therefore, if confounding explains associations in one population, the associations should not be replicated in populations with different confounding structures.

Determining the causality underlying any association is inherently difficult due to the limited ability to measure and control for all possible confounding variables.^8^ First, the study aims to establish whether fetal growth impairment/PTB is an independent and significant risk factor of childhood attention problems and attention deficit hyperactivity disorder (ADHD), after adjustment for multiple maternal, fetal and environmental confounding influences in high-income and middle-income populations. If the association between fetal growth impairment or PTB and child attention problems is entirely due to measured confounding structures, then after full adjustment, no association would be expected. However, even if confounding variables are measured and included in analyses, given the inevitable imprecise measurement of confounding variables, residual confounding is likely.^9^ Second, therefore, the study aims to compare associations across cohorts. If the association between adverse birth outcomes and child attention problems is entirely due to confounding by socioeconomic and healthcare factors, then the associations would be expected to differ between cohorts where these confounding structures are different. Conversely, if the relationships were causal, then associations would be expected to be replicated in cohorts from high-income and middle-income countries, despite the different patterning of socioeconomic and healthcare structures. This method of testing causal inference has been established previously,^10^ ^11^ and has been used to determine causality in the effects of breast feeding^10^ and maternal smoking^11^ on childhood outcomes.

A secondary aim of this study is to determine whether the contribution of factors affecting these associations also vary as a function of symptom severity by comparing associations across two different attentional outcomes which vary both quantitatively (severity) and qualitatively (attention questionnaire vs Diagnostic and Statistical Manual of Mental Disorders, Fourth Edition (DSM-IV) diagnostic criteria). Attention difficulties lie on a continuum from subclinical to clinical-level diagnoses, and brief behavioural questionnaires do not fully capture the entire scope of the clinical disorder.^12^ As such, attention difficulties as identified by a behavioural questionnaire are different, both qualitatively and quantitatively, from clinical-level ADHD, and their aetiologies may also be different. This study therefore used two outcomes for attention problems in childhood, a parental-report measure of child attention difficulties (Strengths and Difficulties Questionnaire (SDQ)^13^), and a clinical-level diagnosis of ADHD based on DSM-IV criteria (Development and Well-Being Assessment (DAWBA)^14^).

## Methods

### The ALSPAC cohort

The ALSPAC cohort consisted of women with an expected delivery date between 1 April 1991 and 31 December 1992, in three health districts in the southwest of England.^15^ A total of 14 541 pregnant women were recruited for the study. Data on birth weight and small-for-gestational age (SGA) status were available for 13 445 children, on head circumference (HC) for 10 397 children and on gestational age at delivery for 13 616 children. Follow-up attention assessments were carried out at approximately 7 years of age (M=7.23 years), and only children with full data available on both attention assessments (SDQ and DAWBA) were included in the final sample (N=6849). Further details of all the data are available through a fully searchable data dictionary at http://www.bris.ac.uk/alspac/researchers/data-access/data-dictionary/. Ethical approval for the study was obtained from the ALSPAC Ethics and Law Committee and Local Research Ethics Committees.

### The 2004 Pelotas cohort

The 2004 Pelotas cohort consisted of children born in 2004, in the city of Pelotas, located in the Brazilian state of Rio Grande do Sul. Pelotas has approximately 340 000 inhabitants, and the major economic activities are agriculture (rice cultivation and cattle farming) and services, including two universities. Mothers residing in the urban area of Pelotas or in the adjacent neighbourhood of Jardim América were recruited and interviewed within 24 h of delivery.^16^ Less than 1% of mothers refused, and a total of 4231 children were successfully recruited into the study. Data on birth weight were available for 4228 children, on SGA for 4218 children, on HC for 4170 children and on gestational age at delivery for 4217 children. Follow-up assessments were carried out when children were approximately 7 years of age^17^ (M=6.70 years), and children with full data available on both attention assessments (SDQ and DAWBA) were included in the final sample (N=3509). The study protocol was approved by the Medical Ethics Committee of the Federal University of Pelotas, affiliated with the Brazilian Federal Medical Council.

### Measures

#### Birth exposures

In both cohorts, neonatal weight and HC were measured within 24 h of delivery by trained researchers. Neonates were classified as low birth weight (LBW) if their birth weight fell under 2500 g,^18^ and as preterm if they were born at <37^+0^ weeks of gestation. SGA was identified using Williams’ curves for birth weight according to gestational age and gender.^19^ Neonates with weight-for-gestational-age below the 10th centile were classified as SGA. Small HC was identified as those neonates whose HC fell below the 10th centile according to the WHO standards.^20^ Information on the technical procedures for obtaining anthropometric measurements and gestational age at birth in both cohorts is presented in online supplementary material.

### Attention outcomes

#### Strengths and Difficulties Questionnaire

The hyperactivity/inattention subscale of the SDQ was used to assess child attention difficulties at a mean age of 6.79 years (SD=0.11) in ALSPAC and 6.70 years (SD=0.19) in Pelotas. The SDQ is a brief, parent-reported, behavioural screening questionnaire for children aged 3–16 years.^13^ It is an extensively evaluated tool^21^ with demonstrated reliability and validity.^22^ The tool was created and validated in English.^13^ Portuguese language translations of the tool have been validated and widely used in Brazilian samples.^23^ ^24^

#### Development and Well-Being Assessment

The DAWBA is a structured set of questions designed to generate DSM-IV psychiatric diagnoses for children aged 5–17 years.^14^ The ADHD subscale of the DAWBA consists of 31 questions, and includes classifications of ‘any ADHD disorder’ as well as specific ADHD subtypes (hyperactive-impulsive, inattentive and combined). The DAWBA was administered to parents of children by questionnaire in ALSPAC at a mean age of 7.66 years (SD=0.14), and by trained interviewers in Pelotas at a mean age of 6.70 years (SD=0.19). The DAWBA has been used extensively as a clinical assessment tool and validated in a Brazilian sample.^25^

### Covariables

Data were collected on a range of maternal, demographic, gestational and perinatal variables in both the ALSPAC and Pelotas cohorts, listed in online supplementary table S1 (online supplementary material). Information on the procedure for obtaining data on all covariables is presented in online supplementary material.

### Statistical analysis

The raw SDQ score was converted to a binary outcome, whereby a score ≥7 indicates attention difficulties.^13^ Cross-cohort differences in the frequency distribution of the birth outcomes (LBW, SGA, small HC and PTB) and attention problems (ADHD and attention difficulties, as measured by the DAWBA and SDQ, respectively) were assessed using Pearson χ^2^ tests. Pearson χ^2^ tests were also used to assess the prevalence of children with attention problems on the SDQ who also had an ADHD diagnosis, and cross-cohort differences in the frequency distribution of covariables, that is, maternal, family, demographic, gestational and perinatal variables.

Logistic regression analyses were conducted to assess the association of covariables with birth outcomes and attention problems in each cohort. For inclusion in the final model, covariables had to be associated with birth outcomes and attention problems at a threshold of p<0.20. Modelling was conducted in the following order, with each successive adjustment including all adjustments in previous models: (1) unadjusted, (2) adjusted for maternal, family and demographic variables and (3) adjusted for gestational and perinatal variables. All models were adjusted for within-cohort variation in child age at the time of testing.

All analyses were performed using IBM SPSS Statistics for Windows V.21.0.^26^

## Results

The prevalence of LBW, SGA, small HC and PTB was approximately twofold higher in Pelotas than in ALSPAC (table 1; see online supplementary table S2, online supplementary material). Attention difficulties, as reported on the SDQ, were higher in Pelotas compared with ALSPAC, as were the inattentive and combined subtypes of ADHD. There was no difference in the frequency of ‘any ADHD disorder’ and ‘hyperactive impulsive ADHD’ between cohorts (table 1). Both cohorts had a similar rate of children who had both attention difficulties (SDQ) and ADHD (DAWBA), 13.5% in ALSPAC and 14.8% in Pelotas (table 2; see online supplementary figure S1, online supplementary material).

**Table 1:** Prevalence of low birth weight, small-for-gestational-age, small HC, preterm birth, ADHD and attention problems in the ALSPAC and Pelotas cohorts

Variable	ALSPAC n (%) (N=6768)	Pelotas n (%) (N=3508)	χ^2^
Exposures			
Low birth weight	238 (3.5)	274 (7.8)	89.99**
SGA	512 (7.6)	496 (14.2)	113.19**
Small HC	264 (4.8)	470 (13.4)	211.02**
Preterm birth	302 (4.4)	445 (12.7)	237.77**
Outcomes			
DAWBA			
Hyperactive-impulsive ADHD	20 (0.3)	13 (0.4)	0.45
Inattentive ADHD	54 (0.8)	11 (0.3)	8.39*
Combined ADHD	68 (1.0)	56 (1.6)	7.13*
Any ADHD disorder	142 (2.1)	92 (2.6)	3.16
SDQ			
Attention difficulties†	719 (10.5)	480 (13.7)	22.94**

*p<0.05; **p<0.001.

†SDQ score ≥7.

ADHD, attention deficit hyperactivity disorder; ALSPAC, Avon Longitudinal Study of Parents and Children; DAWBA, Development and Well-Being Assessment; HC, head circumference; SDQ, Strengths and Difficulties Questionnaire; SGA, small-for-gestational age.

**Table 2:** Prevalence of children with attention difficulties on the SDQ with ADHD diagnoses (DAWBA)

Variable	ADHD	No ADHD	χ^2^
ALSPAC† (n=719)	97 (13.5)	622 (86.5)	515.81**
Pelotas† (n=480)	71 (14.8%)	409 (85.2)	322.57**

*p<0.05; **p<0.001.

†Attention problems=SDQ score ≥7.

ADHD, attention deficit hyperactivity disorder; ALSPAC, Avon Longitudinal Study of Parents and Children; DAWBA, Development and Well-Being Assessment; SDQ, Strengths and Difficulties Questionnaire.

To assess cross-cohort differences in the confounding structures, the frequency distribution of covariables was compared, as was the association between covariables and each of the exposures and outcomes (see online supplementary table S1; online supplementary material). The Pelotas cohort had a higher prevalence of mothers with a low level of education, a higher rate of low income, more mothers aged <19 years at delivery, a higher prevalence of smoking during pregnancy, a higher prevalence of depression during pregnancy, a higher rate of caesarean delivery and a higher prevalence of babies with a 5 min Apgar score <7. There were also differences in the associations between covariables and both birth exposures and attention problems, particularly in relation to caesarean sections (see online supplementary table S3; online supplementary material). In ALSPAC, caesarean section was more strongly associated with each of the adverse birth outcomes compared with Pelotas. Furthermore, there was an opposite effect of caesarean sections on attention problems across cohorts, whereby caesarean section was associated with an increased risk of attention problems in ALSPAC, but a decreased risk in Pelotas (table 3).

**Table 3:** ORs (95% CI) of ADHD or attention difficulties as measured by the DAWBA and SDQ in the ALSPAC and Pelotas cohorts

	Any ADHD disorder (DAWBA)		Hyperactivity (SDQ)	
Variable	ALSPAC	Pelotas	ALSPAC	Pelotas
Maternal education†	1.03 (0.68 to 1.55)	1.96 (1.16 to 3.29)*	1.45 (1.21 to 1.73)**	1.74 (1.39 to 2.19)**
Income (lowest quintile vs rest)	1.05 (0.51 to 2.17)	1.15 (0.70 to 1.90)	1.68 (1.26 to 2.25)**	1.29 (1.02 to 1.62)*
Maternal age at delivery (≤19 vs rest)	2.38 (1.03 to 5.50)*	1.17 (0.71 to 1.94)	2.22 (1.43 to 3.43)**	1.63 (1.21 to 2.04)**
Maternal smoking during pregnancy (yes vs no)	1.54 (1.05 to 2.27)*	1.24 (0.80 to 1.94)	1.85 (1.55 to 2.21)**	1.38 (1.12 to 1.70)*
Maternal alcohol during pregnancy (yes vs no)	1.10 (0.76 to 1.58)	2.08 (0.89 to 4.86)	1.16 (0.98 to 1.38)	1.00 (0.59 to 1.71)
Depression during pregnancy (yes vs no)	1.81 (1.18 to 2.78)*	1.83 (1.19 to 2.81)*	1.82 (1.48 to 2.23)**	1.42 (1.15 to 1.76)*
Apgar score 5 min (<7 vs rest)	1.05 (0.14 to 7.73)	1.42 (0.34 to 5.92)	0.89 (0.31 to 2.51)	1.78 (0.93 to 3.40)
Mode of delivery (caesarean vs vaginal)	1.54 (0.94 to 2.52)	0.79 (0.51 to 1.20)	1.15 (0.89 to 1.48)	0.81 (0.67 to 0.99)*

*p<0.05; **p<0.001.

†≤10 vs >10 years.

ADHD, attention deficit hyperactivity disorder; ALSPAC, Avon Longitudinal Study of Parents and Children; DAWBA, Development and Well-Being Assessment; SDQ, Strengths and Difficulties Questionnaire.

Overall, the pattern of associations between birth outcomes and attention difficulties, as measured by the SDQ, was similar between ALSPAC and Pelotas across all adjustments, with stronger associations for LBW, SGA, and small HC, than for PTB (table 4). In unadjusted models, attention difficulties were associated with LBW (OR=1.66), SGA (OR=1.71), small HC (OR=1.92) and PTB (OR=1.44) in ALSPAC, and SGA (OR=1.47) and small HC (OR=1.31) in Pelotas. Following full adjustment for maternal, family, demographic, gestation and perinatal variables, associations remained between SGA (OR=1.59) and small HC (OR=1.64) in ALSPAC and SGA (OR=1.35) in Pelotas.

**Table 4:** Unadjusted and adjusted associations between birth anthropometric measures and ADHD/attention difficulties in ALSPAC and Pelotas cohorts

Variable	Unadjusted*			Model 1†			Model 2‡		
	OR	95% CI	p Value	OR	95% CI	p Value	OR	95% CI	p Value
*Attention difficulties (SDQ)*									
ALSPAC									
Low birth weight	1.66	1.16 to 2.36	0.005	1.69	1.17 to 2.42	0.005	1.46	0.98 to 2.17	0.060
Small-for-gestational age	1.71	1.33 to 2.19	<0.001	1.62	1.23 to 2.14	0.001	1.59	1.20 to 2.19	0.001
Small head circumference	1.92	1.39 to 2.67	<0.001	1.73	1.20 to 2.50	0.003	1.64	1.11 to 2.41	0.012
Preterm birth	1.44	1.03 to 2.01	0.031	1.44	1.03 to 2.01	0.031	1.37	0.95 to 1.98	0.094
Pelotas									
Low birth weight	1.35	0.97 to 1.88	0.077	1.31	0.94 to 1.83	0.114	1.21	0.86 to 1.71	0.287
Small-for-gestational age	1.47	1.14 to 1.89	0.003	1.43	1.10 to 1.84	0.007	1.35	1.04 to 1.75	0.023
Small head circumference	1.31	1.00 to 1.71	0.047	1.32	1.01 to 1.72	0.043	1.17	0.89 to 1.54	0.256
Preterm birth	1.10	0.83 to 1.46	0.513	1.03	0.77 to 1.37	0.848	0.99	0.74 to 1.32	0.938
*Any ADHD disorder (DAWBA)*									
ALSPAC									
Low birth weight	2.13	1.11 to 4.11	0.024	2.13	1.11 to 4.12	0.024	2.29	1.09 to 4.80	0.029
Small-for-gestational age	0.83	0.42 to 1.63	0.583	0.83	0.42 to 1.63	0.583	0.65	0.30 to 1.41	0.275
Small head circumference	1.05	0.46 to 2.40	0.917	1.05	0.46 to 2.40	0.917	1.06	0.43 to 2.65	0.896
Preterm birth	2.23	1.24 to 3.99	0.007	2.23	1.24 to 3.99	0.007	2.33	1.23 to 4.42	0.009
Pelotas									
Low birth weight	0.83	0.36 to 1.92	0.668	0.81	0.35 to 1.87	0.618	0.78	0.34 to 1. 80	0.560
Small-for-gestational age	1.77	1.07 to 2.94	0.027	1.71	1.03 to 2.84	0.038	1.69	1.02 to 2.82	0.043
Small head circumference	0.91	0.48 to 1.72	0.764	0.87	0.46 to 1.65	0.671	0.85	0.45 to 1.62	0.630
Preterm birth	0.95	0.50 to 1.79	0.863	0.90	0.48 to 1.71	0.754	0.87	0.46 to 1.65	0.664

*Adjusted for age at time of testing.

†Adjusted for age at time of testing and maternal, family and demographic variables (maternal education, income, maternal age at delivery).

‡Adjusted for model 1 and gestational (smoking, alcohol use, and depression during pregnancy) and perinatal variables (mode of delivery and Apgar score at 5 min).

ADHD, attention deficit hyperactivity disorder; ALSPAC, Avon Longitudinal Study of Parents and Children; DAWBA, Development and Well-Being Assessment; SDQ, Strengths and Difficulties Questionnaire.

The pattern of associations between birth outcomes and ADHD differed markedly between the ALSPAC and Pelotas cohorts, with opposing directions of association for all birth outcomes, across all adjustments (table 4). In unadjusted models, ADHD was associated with LBW (OR=2.13) and PTB (OR=2.23) in ALSPAC but not in Pelotas (OR=0.83 and OR=0.95 respectively), and with SGA (OR=1.77) in Pelotas, but not in ALSPAC (OR=0.83). Following adjustment for maternal, family, demographic, gestational and perinatal variables, ADHD was associated with LBW (OR=2.29) and PTB in ALSPAC (OR=2.33) but not in Pelotas (OR=0.78 and OR=0.87, respectively). ADHD was associated with SGA (OR=1.69) in Pelotas but not in ALSPAC (OR=0.65).

## Discussion

The primary aim of this study was to help improve causal inference in the associations between adverse birth outcomes and attention impairment in childhood. Associations were identified between SGA and small HC, and attention difficulties as identified by the SDQ, and the pattern of associations was similar between the ALSPAC and Pelotas cohorts. This similar patterning across cohorts suggests that these associations are unlikely to be an artefact of confounding structures, and may be causal. The secondary aim of the study was to determine whether the aetiology of child attention problems may differ between two measures that capture different ends of the attention impairment spectrum (parent-report attention difficulties vs clinical-level diagnoses). After controlling for covariables, the association between SGA and parent-reported attention difficulties at 7 years of age remained significant in both ALSPAC and Pelotas. No such pattern was identified for ADHD between cohorts, however, with associations in the opposite directions for all birth outcomes. As such, whereas being born SGA or with a small HC might play a causal role in the later development of attention difficulties, other factors may affect the more extreme cases of ADHD diagnosis.

Our primary findings are consistent with previous studies that have identified SGA^27^ ^28^ and small HC^1^ ^29^ as a predictor of attention problems in childhood. Being born SGA or with a low HC are related to impaired fetal growth, the physiological consequences of which also affect neural development.^30^ Neurostructural studies in human and animals being born SGA or with small HC have identified decreases in cortical grey matter, axon myelination, dendritic arborisation, synaptogenesis and neurotransmitter availability.^31–33^ Such neurostructural impairments may provide an explanation for the causal association between being born SGA or with small HC and attention difficulties in childhood.

Our secondary findings showed that the pattern of association for ADHD differed significantly between cohorts while that of attention problems (SDQ) did not. These results indicate that confounding structures may have a greater effect on ADHD diagnoses than they do on milder attention difficulties. Even though milder attention difficulties and ADHD diagnoses both lie on the continuum of attention impairment, ADHD diagnoses represent a greater severity of impairment, compared with subclinical attention difficulties. Increased severity of impairment may arise from the accumulation of a greater number of risk factors, compared with subclinical attention difficulties, thus resulting in different aetiological profiles. The results of this study suggest that while impaired fetal growth may predispose a child to attention difficulties, whether their level of impairment reaches the threshold for ADHD diagnosis may be a function of environmental influences such as familial and childhood factors. This has been supported by a number of studies and reviews which have suggested that environmental influences in childhood, such as psychosocial adversity (including low-income, in-home discord and parenting practices)^34^ ^35^ and early traumatic events,^36^ may contribute to additional risk for the development of ADHD.

### Strengths and limitations

This study compared cohorts from a high-income and middle-income country as a method to examine causal inference in the associations between adverse birth outcomes and attention problems in childhood. Both cohorts were well established, used the same outcome measures at comparable time points in childhood, and have a range of sociodemographic, gestational and perinatal information, allowing for identical adjustment methods across both cohorts and minimising statistical heterogeneity. The advantage of comparing cohorts from high-income and middle-income countries is that there are expected to be systematic differences in confounding structures, which may impact the exposures and/or outcomes differently. For example, in this study, marked differences were identified between the ALSPAC and Pelotas cohorts with regard to the proportion and effects of caesarean delivery. In ALSPAC, caesarean delivery was associated with being born LBW, SGA or preterm; however there was no increased risk in Pelotas. In fact, caesarean delivery decreased the risk of being born with a small HC in Pelotas.

These findings reflect marked differences between the UK and Brazil regarding elective caesarean sections. Whereas Brazil has one of the highest rates of caesarean section in the world,^37^ with approximately 1 000 000 elective caesareans per year, the UK has only approximately 50 000.^38^ This large variation reflects the social and cultural approach to obstetric care in Brazil.^39^ In Brazil, elective caesarean delivery is associated with higher maternal education, income and urban dwelling,^37^ ^40^ associations which are not evident in the UK.^41^ As such, comparing cohorts with such marked differences in the effect of maternal and obstetric care practices provides an opportunity to disentangle the causal role of adverse birth outcomes and attention impairment in childhood.

There are some limitations which must be acknowledged. First, although within-cohort variability in age at the time of testing was adjusted for in all models, children in the ALSPAC cohort were assessed on the DAWBA slightly older (7 years, 7 months) than in the Pelotas cohort (6 years, 8 months). It is unlikely, however, that this 11-month difference would cause a significant impact on the outcome, as ADHD symptoms have been shown to be relatively persistent across this time period.^42^ Second, characteristic of prospective birth cohort studies, there was attrition of participants during the follow-up period. Examination of differences between the sample and losses, however, showed that the differences between sample and loss groups were systematic across both cohorts. In ALSPAC and Pelotas, the loss group had a higher rate of LBW, SGA, small HC and PTB, which is likely to reflect the higher rate of neonatal death among the loss group. Furthermore, when the entire cohort was included in analyses, rather than restricting the sample to just those who had completed the follow-up, the direction and magnitude of associations between birth outcomes and covariables were similar in both cohorts. This suggests that the samples in this study were not systematically different from the full cohorts in the associations between covariables and birth outcomes, and can thus be considered representative of the full cohort. Finally, while cross-cohort comparison improves causal inference, it is important to note that this approach only separates causal effects from confounding structures which differ between cohorts. Therefore, it remains possible that the consistent associations found between fetal growth variables and attentional problems across cohorts are explained by covariables which are also consistent across cohorts (eg, genetic and epigenetic confounding).

### Implications and conclusions

The findings of this study suggest some implications for the clinical management of children with disturbed fetal growth, for the guidance of education provisions for children with attention impairment, and for the direction of epidemiological and clinical research into factors associated with ADHD diagnosis. Effective interventions during early childhood for small HC/SGA children may promote early neurodevelopment, and reduce academic failure and behavioural difficulties at the onset of formal schooling.^43^ Further research is required to examine other factors that may be contributing to the likelihood of receiving a diagnosis of ADHD to ensure early and precise identification of vulnerable children.

In conclusion, this study identified that fetal growth impairment may play a causal role in the development of attention difficulties in childhood, but that confounding structures may play a greater role in whether a child meets the full diagnostic criteria for ADHD. These findings have important implications for the clinical management of children with growth impairment during fetal life, where early intervention may buffer the effect of subclinical attention difficulties on educational outcomes.

**What is already known on this subject**

- Associations between fetal growth impairment or preterm birth and attention problems in childhood have been well established, although it remains unclear whether these associations may be an artefact of residual confounding by social, health and economic factors. Cross-cohort comparison provides a method for improving causal inference in these associations by examining whether associations differ between cohorts with different confounding structures.

**What this study adds**

- This study identified that fetal growth impairment may play a causal role in the development of attention difficulties in childhood; however, social, health and economic factors may play a greater role in whether a child meets the full diagnostic criteria for attention deficit hyperactivity disorder (ADHD). Targeted early intervention programmes in early childhood may enhance attention development of children with fetal growth impairment, and buffer the effect of subclinical attention difficulties on educational outcomes.

## Supplementary Material

Web supplement
