# Supplementary material for: Are fetal growth impairment and preterm birth causally related to child attention problems and ADHD? Evidence from a comparison between high-income and middle-income cohorts
Source: J Epidemiol Community Health. 2016 Jan 14;70(7):704–9. doi: 10.1136/jech-2015-206222 (PMC4941187; doi:10.1136/jech-2015-206222)
Supplement: Web supplement [file jech-2015-206222-s1.pdf]

**Are fetal growth impairment and preterm birth causally related to child attention problems and ADHD? Evidence from a comparison between high- and middle-income cohorts.**

Elizabeth Murray, Rebecca Pearson, Michelle Fernandes, Iná S. Santos, Fernando C. Barros, Cesar G. Victora, Alan Stein, Alicia Matijasevich

**Supplemental Materials**

**Word count:** 491 words

**Number of tables:** 3

**Number of figures:** 1

**Supplemental Methods**

## Birth outcome data collection

*ALSPAC*

Birthweight was recorded from the routine birth record. Head circumference was measured using a Holtain neonatometer (Holtain Ltd., Crymlych, Wales) by trained ALSPAC staff who visited all study participants within 24 hours of birth. Gestational age was measured from the date of the last menstrual period, provided it was in agreement with the results of the dating ultrasound scan (<20 weeks). In case of a discrepancy between the stated date of the last menstrual period and the date identified by ultrasound, estimation was made by an experienced obstetrician.

*Pelotas*

Maternity staff weighed newborns and other anthropometric measures were conducted by trained and supervised study interviewers at the time of birth. Newborns were weighed using regularly calibrated pediatric scales, and length measurements were taken in supine position using ARTHAG infantometers with 1mm precision. Head circumference was measured by standardized tape measure. Gestational age was estimated using the algorithm proposed by the National Center for Health Statistics (NCHS)[1], based on the last menstrual period, when consistent with birthweight, length and head circumference (HC)[2]. If inconsistencies were identified, or if the date of last menstrual period was unknown, then gestational age was determined by clinical maturity estimate, based on the Dubowitz method[3].

## Covariable data collection

*ALSPAC*

Data on potential confounders were collected in self-report postal questionnaires completed by the mother during pregnancy or in the neonatal period. In ALSPAC, the highest level of maternal schooling was assessed by maternal self-report in a postal questionnaire completed at approximately 32 weeks gestation. Mothers were dichotomised into two groups with a cut-off point approximately equivalent to ten years of schooling (non-O level (GSCE) vs O level and above). Family income was postnatally assessed by questionnaire at 47 months (categorised into five groups: <£100; £100-199; £200-299; £300-399; >£400 per week). Data on maternal depression was collected at 32 weeks gestation using the Edinburgh Postnatal Depression Scale (EPDS)[4]. Mothers were classified as having depression during pregnancy if they had an EPDS score ≥13[4]. Maternal age was collected at approximately 12 weeks gestation. Data on maternal education, depression during pregnancy (Edinburgh Postnatal Depression Scale; EPDS[4]), smoking and alcohol consumption was collected at 32 weeks gestation. Obstetric information regarding mode of delivery and APGAR score[5] at 5 minutes was collected from obstetric records at birth.

*Pelotas*

Data on maternal age, maternal smoking during pregnancy, maternal alcohol consumption during pregnancy, maternal schooling, family income and maternal mental health during pregnancy were

collected in interview format in the perinatal period. Family income was based on maternal report one month prior to delivery, and was classified into quintiles. Maternal number of school years was collected in the perinatal interview, and dichotomised into two groups (≤10 years vs >10 years of schooling). Maternal smoking habits, alcohol consumption, and depression during pregnancy were based on maternal report, and assessed retrospectively at birth. Child characteristics collected in the perinatal interview included data on mode of delivery, and APGAR score[5] at five minutes.

**Table S1**

Prevalence of confounding factors in the ALSPAC and Pelotas cohorts

| Variable | ALSPAC n (%) (N=6768) | Pelotas n (%) (N=3508) | $\chi^2$ |
|---|---|---|---|
| **Family and maternal** | | | |
| Low maternal education[a] | 1471 (21.9) | 2367 (68.1) | 2082.30** |
| Low income | 379 (6.4) | 685 (19.5) | 375.50** |
| Maternal age at delivery ≤19 years | 128 (1.9) | 674 (6.5) | 976.70** |
| **Gestational** | | | |
| Smoked during pregnancy | 1273 (18.9) | 952 (27.1) | 91.66** |
| Any alcohol during pregnancy | 4345 (65.7) | 117 (3.3) | 3614.29** |
| Maternal depression during pregnancy | 836 (12.7) | 538 (15.8) | 18.11** |
| **Perinatal variables** | | | |
| Caesarean delivery | 652 (9.8) | 1574 (44.9) | 1640.46** |
| APGAR score <7 at 5 minutes | 38 (0.9) | 55 (1.6) | 6.35* |

[a] ≤10 years education; *p<.05; **p<.001

**Table S2**

Subgroups of gestational age at birth in the ALSPAC and Pelotas cohorts

| **Variable** | **ALSPAC n (%) (N=6848)** | **Pelotas n (%) (N=3505)** | $\chi^2$ |
|---|---|---|---|
| ≤32 weeks | 53 (0.8) | 57 (1.6) | 241.23** |
| 33-34 weeks | 53 (0.8) | 82 (2.3) | |
| 35-36 weeks | 196 (2.9) | 306 (8.7) | |
| ≥37 weeks | 6546 (95.6) | 3060 (87.3) | |

*$p$<.05; **$p$<.001

**Table S3**

Odds Ratios (95% CI) of low birth weight, SGA, small HC and low PI in the ALSPAC and Pelotas cohorts

| **Variable** | **Low Birth Weight** | | **SGA** | | **Small HC** | | **Preterm birth** | |
|---|---|---|---|---|---|---|---|---|
| | **ALSPAC** | **Pelotas** | **ALSPAC** | **Pelotas** | **ALSPAC** | **Pelotas** | **ALSPAC** | **Pelotas** |
| Maternal education[a] | 1.22 (0.90-1.66) | 1.27 (0.96-1.68) | 1.25 (1.01-1.54)* | 1.29 (1.04-1.59)* | 1.28 (0.96-1.70) | 1.40 (1.12-1.74)* | 1.07 (0.81-1.42) | 1.43 (1.14-1.80)* |
| Income (lowest quarter vs rest) | 1.35 (0.81-2.24) | 1.79 (1.36-2.36)** | 1.40 (0.98-2.00) | 1.29 (1.03-1.63)* | 1.55 (0.98--2.44) | 1.67 (1.33-2.08)** | 0.85 (0.59-1.47) | 1.73 (1.38-2.16)** |
| Maternal age at delivery (≤19 vs rest) | 1.63 (0.75-3.54) | 1.20 (0.89-1.61) | 1.17 (0.63-2.19) | 1.07 (0.85-1.36) | 1.31 (0.57-3.01) | 1.48 (1.18-1.87)* | 0.88 (0.36-2.17) | 1.54 (1.22-1.94)** |
| Maternal smoking during pregnancy (yes vs no) | 1.76 (1.31-2.36)** | 1.66 (1.29-2.15)** | 2.55 (2.10-3.10)** | 1.69 (1.39-2.07)** | 1.56 (1.17-2.08)* | 1.63 (1.32-2.00)** | 1.02 (0.76-1.38) | 1.23 (0.99-1.53) |
| Maternal alcohol during pregnancy (yes vs no) | 0.86 (0.65-1.12) | 0.98 (0.49-1.96) | 0.87 (0.72-1.05) | 1.11 (0.66-1.85) | 0.86 (0.66-1.11) | 1.18 (0.71-1.96) | 0.84 (0.66-1.08) | 1.43 (0.88-2.35) |
| Depression during pregnancy (yes vs no) | 1.84 (1.31-2.60)* | 1.37 (1.04-1.80)* | 1.11 (0.85-1.46) | 1.19 (0.56-1.47) | 1.69 (1.23-2.33)* | 1.22 (0.98-1.52) | 1.70 (1.24-2.31)* | 1.35 (1.08-1.68)* |
| APGAR score 5 min (<7 vs rest) | 4.82 (2.09-11.13)** | 7.28 (4.14-12.80)** | 0.98 (0.30-3.20) | 1.97 (1.05-3.69)* | 2.47 (0.86-7.09) | 2.77 (1.50-5.10)* | 5.74 (2.75-11.95)** | 4.55 (2.61-7.94)** |
| Mode of delivery (caesarean vs vaginal) | 2.79 (1.94-3.94)** | 1.05 (0.82-1.35) | 1.45 (1.10-1.91)* | 0.86 (0.71-1.04) | 1.30 (0.99-1.92) | 0.78 (0.64-0.95)* | 2.09 (1.51-2.90)** | 1.03 (0.84-1.25) |

[a] ≤10 years vs >10 years; **p*<.05; ***p*<.001

**Figure S1.** Descriptive statistics of SDQ score based on ADHD diagnosis in ALSPAC and Pelotas

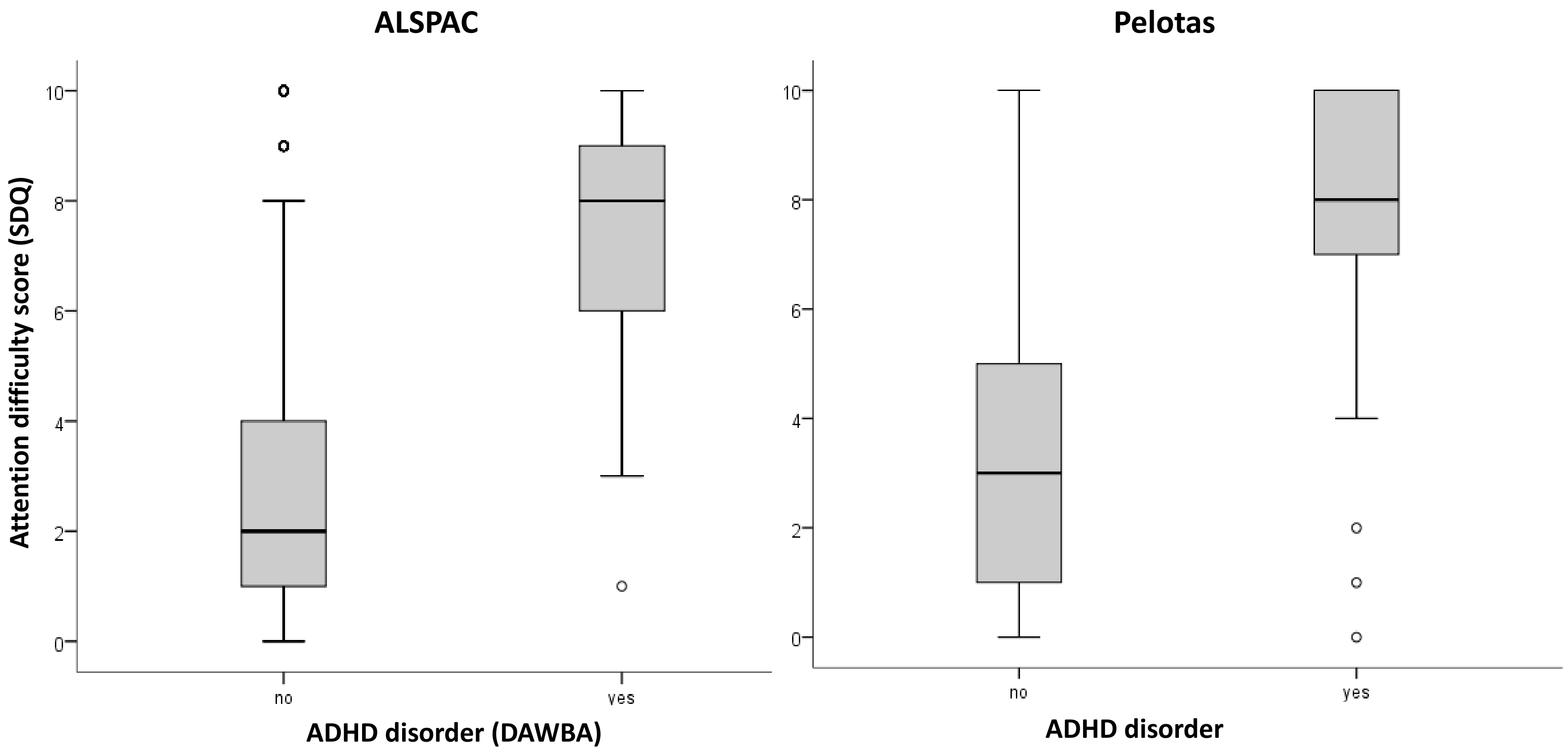

## References


1. Taffel S, Johnson D, Heuser R. A method of imputing length of gestation on birth certificates. 1982.
2. Barros AJ, Santos IdSd, Victora CG, et al. The 2004 Pelotas birth cohort: methods and description. *Revista de saude publica.* 2006;40(3):402-413.
3. Dubowitz L, Dubowitz V, Goldberg C. Clinical assessment of gestational age in the newborn infant. *The Journal of pediatrics.* 1970;77(1):1-10.
4. Cox JL, Holden JM, Sagovsky R. Detection of postnatal depression. Development of the 10-item Edinburgh Postnatal Depression Scale. *The British journal of psychiatry.* 1987;150(6):782-786.
5. Apgar V. A proposal for a new method of evaluation of the newborn. *Curr Res Anaesth.* 1953;32:260-267.
